# Characterization of the *Plasmodium vivax* erythrocytic stage proteome and identification of a potent immunogenic antigen of the asexual stages

**DOI:** 10.1186/1475-2875-9-S2-P44

**Published:** 2010-10-20

**Authors:** Wanlapa Roobsoong, Sittiruk Roytrakul, Rachada Kiatfuengfoo, Wilairat Nuchpramool, Liwang Cui, Rachanee Udomsangpetch

**Affiliations:** 1 Faculty of Medical Technology, Mahidol University, Bangkok 10700, Thailand; 2 National Center for Genetic Engineering and Biotechnology, Pathumthani 12120, Thailand; 3 Department of Entomology, Penn State University, University Park, Pennsylvania 16801, USA; 4 Department of Pathobiology, Faculty of Science, Mahidol University, Bangkok 10400, Thailand

## Background

With the genome of *Plasmodium vivax* sequenced [1], it would be important to determine proteomes of the parasite in order to assist efforts in understanding the basic biology of the parasite as well as provides the new tools for identifying novel antigens and drug targets.

## Materials and methods

Lysates of *P. vivax* were separated by SDS-polycrylamide gel electrophoresis (SDS-PAGE) and proteins were identified by using matrix-assisted laser desorption/ionization-time of flight (MALDI-TOF/TOF) mass spectrometry. In addition, to identify proteins that might be recognized by host humoral immunity, we have separated them by two-dimensional gel electrophoresis. Proteins were then screened by western blot with immune serum. Spots that were recognized by the host serum were excised and identified by high-accuracy liquid chromatography-tandem mass spectrometry (LC-MS/MS).

## Results

**Figure 1:**
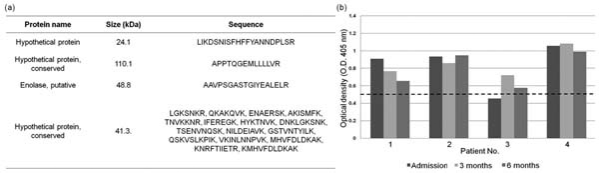
*Plasmodium vivax* proteins recognized by immune serum (a), and antibody responses to PV180L protein in convalescence sera (dash line indicate ELISA cut-off value).

Several hundred proteins were confidently identified. All proteins were classified into functional classes (see Table 1). Four parasite proteins were recognized by *P. vivax*-immune human sera. Interestingly, one of the four proteins (PV180L), reacted with the convalescence sera, 6 months post treatment of *P. vivax*-immune donors (see Figure 1).

**Table 1:** Functional classes of all identified proteins.

Functional classes	%
Cellular transport	4.0
Hypothetical	48.0
Metabolism	4.0
Protein fate	5.0
Protein synthesis	4.0
Protein with binding function	10.0
Unclassified	18.0
Others	7.0

## Conclusion

The PV180L encodes a 24.1 kDa hypothetical protein which expressed throughout the erythrocytic cycle of *P. vivax* and the antibodies to PV180L were long lasting. Therefore, more reports on the proteins of *P. vivax* parasite provide useful information and availability to facilitate not only basic research on this extraordinary malaria parasite, but also provide the new tools for drug and vaccine developments.
